# RER1 enhances carcinogenesis and stemness of pancreatic cancer under hypoxic environment

**DOI:** 10.1186/s13046-018-0986-x

**Published:** 2019-01-10

**Authors:** Shi Chen, Jiaqiang Zhang, Jiangzhi Chen, Yaodong Wang, Songqiang Zhou, Long Huang, Yannan Bai, Chenghong Peng, Baiyong Shen, Huixing Chen, Yifeng Tian

**Affiliations:** 1 Department of Hepatobiliary Surgery, Fujian Provincial Hospital, Fujian Medical University, Fuzhou, 350001 People’s Republic of China; 2 Department of Pancreatic Surgery, Ruijin Hospital, Shanghai Jiaotong University School of Medicine, Shanghai, 200025 People’s Republic of China; 3 Department of Hepatobiliary Surgery, Union Hospital, Fujian Medical University, Fuzhou, 350001 People’s Republic of China

**Keywords:** Pancreatic cancer (PC), Retention in endoplasmic reticulum 1 (RER1), Hypoxia, Stemness, Hypoxia-inducible factor (HIF)-1α

## Abstract

**Background:**

Increasing incidence and mortality rates of pancreatic cancer (PC) highlight an urgent need for novel and efficient drugs. Retention in endoplasmic reticulum 1 (RER1) is an important retention factor in the endoplasmic reticulum (ER). However, it remains elusive whether RER1 is involved in the retention of disease-related proteins.

**Methods:**

We analyzed the expression level of RER1 in PC and adjacent tissues, and also employed Kaplan–Meier’s analysis to identify the correlation between RER1 expression and overall survival rate. Cell proliferation, colony formation, tumor formation, scratch test, and transwell invasion assays were performed in RER1 knockdown cells and negative control cells.

**Results:**

We hereby reported the important functions of RER1 in tumorigenesis and metastasis of PC, evidenced by inhibitory effects of RER1 knockdown on PC cell proliferation, migration and aggressiveness. Tumor formation was also significantly repressed in RER1 knockdown cells compared to control. Hypoxia-inducible factor (HIF)-1α was found to be an upstream regulator of RER1. Knockdown HIF-1α cells exhibited similar repressive impact on cell proliferation as RER1, and showed diminished migratory and invasive abilities under hypoxic condition.

**Conclusion:**

The present study has demonstrated that RER1 enhances the progression of PC through promoting cell proliferation, migration and aggressiveness.

## Introduction

With the developments in early detection, prevention and treatment of cancer, most cancers have showed a steadily declining incidence over the past several decades, with the exception of pancreatic cancer (PC). PC has shown an increasing trend, and the 5-year survival rate is only 8% as one of the leading causes of cancer death [1–3], mainly due to its late presentation, low surgical resectability rate, limited treatment, resistance to standard radiotherapy and chemotherapy, and recurrence [4]. Therefore, it is an urgent need to understand the molecular mechanism underlying PC tumorigenesis and to identify new therapeutic targets.

One possible explanation for the high mortality rate of PC is the existence of cancer stem cells (CSCs) [5, 6]. These cells possess self-renewal ability and are capable of differentiation, tumorigenicity and metastasis [5, 7]. It has been hypothesized that CSCs are the clonogenic core of tumors [8]. However, most therapies target only tumor cells, allowing the escape of CSC population residing within the tumor causing the relapse of cancer resistant to chemotherapy and radiotherapy. Thus, due to their important roles in cancer development and relapse, CSCs are deemed potentially efficient targets for cancer treatments.

Retention in endoplasmic reticulum 1 (RER1) was first identified in yeast and functions as an important retention factor in the endoplasmic reticulum (ER) for several proteins, including Sec12p, Sed4p, Mns1p, Sec71p and Sec63p [9–12]. Yeast Rer1 localizes to the cis-Golgi, where it interacts with numerous proteins and recruits them to coat protein I vesicles to translocate them into the ER [13]. Previous evidence showed that human RER1 has similar function as yeast Rer1, because it was found to complement the defective phenotype observed in the Rer1 deleted yeast strain [12]. Interestingly, a previous study demonstrated that RER1 played a crucial role in Notch signaling activation, which regulated mouse cerebral cortex development [14]. It is known that Notch signaling is an important regulator of PC CSCs [15]. Therefore, we hypothesized RER1 was likely involved in PC CSCs. To test this hypothesis, we investigated whether RER1 promoted the progression of PC and if RER1 played a role in PC CSCs. It was found that RER1 expression was higher in PC tissues than in adjacent normal tissues. RER1 knockdown significantly repressed PC cell proliferation, migration, aggressiveness and tumor formation. It was revealed that hypoxia-inducible factor (HIF)-1α specifically regulated RER1, and knockdown HIF-1α cells exhibited similar repressive impact as RER1 on cell proliferation, and showed diminished migratory and invasive abilities under hypoxic condition.

## Methods

### Cell culture

Six PC cell lines (AsPC-1, Mia PaCa-2, SW1990, Capan-2, CFPAC-1 and PANC-1) and a normal human pancreatic ductal cell line (hTERT-HPNE, CRL-4023) were purchased from the American Type Culture Collection (ATCC, Manassas, VA, USA). All the PC cells were cultured in Dulbecco’s Modified Eagle Medium (DMEM)/F12 (Sigma, St Louis, MO, USA) supplemented with 10% fetal bovine serum (FBS; Invitrogen, Carlsbad, CA, USA) and 1% penicillin-streptomycin (Gibco, Grand Island, NY). hTERT-HPNE cell line was cultured according to the method recommended by ATCC (Rockville, MD, USA), in 75% DMEM without glucose (Sigma Cat#. D-5030 with additional 2 mM L-glutamine and 1.5 g/L sodium bicarbonate) and 25% Medium M3 Base (Incell Corp. Cat# M300F- 500) supplemented with 5% FBS, 10 ng/mL human recombinant epidermal growth factor, 5.5 mM D-glucose (1 g/L) and 750 ng/mL puromycin. All cells were incubated at a humidified atmosphere supplied with 5% CO_2_ at 37 °C.

### Knockdown and overexpression of RER1

Knockdown of RER1 was achieved using RER1 shRNAs (sh-RER1–1 and sh-RER1–2) as previously described [16], using an empty vector (sh-CTR) as control. The sequences of RER1 shRNAs were shown below: RER1 shRNA1: F: ccggGACTTGGACAGATTTATCAGTggatccACTGATAAATCTGTCCAAGTCtttttg, R: aattcaaaaaGACTTGGACAGATTTATCAGTggatccACTGATAAATCTGTCCAAGTC; RER1 shRNA2: F: ccggGGCCGATTCTGGTGATGTACTggatcc AGTACATCACCAGAATCG GCCtttttg, R: aattcaaaaaGGCCGATTCTGGTGATGTACTggatccAGTACATCACCAGAATCGGCC. Overexpression of RER1 was achieved by transfection of RER1 plasmid (pSin-RER1), using an empty vector (pSin-vec) as control. The primers used to clone RER1 were: F: CTAGAATTCATGTCTGAAGGGGACAGTG, R: CTAGGATCCCTAGCTGGCGAAGGCCTTGCCG. Stable knockdown and overexpression of RER1 were obtained using viral infection. Firstly, the RER1 shRNA plasmids and pSin-RER1 were transfected together with gag/pol, rev and VSVG plasmids to HEK293T cells with the ratio of 2/2/2/1. After collection from HEK293T cells, the virus was used to infect cells followed by puromycin selection to establish the culture of only positively infected cells with stable RER1 knockdown. Infection efficiency was estimated to be approximately 80% based on observations made on the culture during puromycin selection.

### Immunohistochemistry staining

Paraffin-embedded sections were deparaffinized in xylene and rehydrated in a graded series of ethanol. Antigen retrieval was achieved using sodium citrate buffer (10 mM sodium citrate, 0.05% Tween 20, pH 6.0). Slides were boiled by a cooker for 10 min, and were then cooled to room temperature. The activity of endogenous peroxidases was blocked by 3% hydrogen peroxide in methanol for 20 min, followed by washing using tris-buffered saline (TBS) with 0.025% Triton X-100 for 5 times. Then the slides were blocked using 10% normal serum with 1% BSA in TBS for 2 h at room temperature. RER1 antibody 100-fold diluted (R4407, Sigma, St. Louis, MO) or anti-Ki67 (#ab16667; Abcam, Cambridge, MA, USA) were used to specifically recognize the antigens on slides, which were incubated with slides overnight at 4 °C followed by immunostaining with a secondary antibody (#191866, Abcam, Cambridge, MA, USA, 0.2 μg/ml) at 37 °C for 1 h. Afterwards, all slides were incubated with streptavidin-horseradish peroxidase (HRP) conjugate complex at 37 °C for 1 h and developed with 3,3′-diaminobenzidine.

### Western blot analysis

Protein extracts were obtained by radioimmunoprecipitation assay buffer (Invitrogen, Waltham, MA USA) containing 25 mM Tris-HCl pH 7.6, 150 mM NaCl, 1% NP-40, 1% sodium deoxycholate and 0.1% SDS. Equivalent amounts of protein extracts were separated using SDS-PAGE and transferred to polyvinylidene difluoride membrane (Sigma-Aldrich, St. Louis, MO). After blocking the membranes with 8% fat-free milk (in TBS with 0.1% Tween 20) for 1 h at 37 °C, primary antibodies were applied as 1:1000 including anti-RER1 (Sigma, St. Louis, MO), anti-E-cadherin (24E10, Cell Signaling Technology, Danvers, MA, USA), anti-N-cadherin (clone GC-4, Sigma, St. Louis, MO), anti-vimentin (RV202, Abcam, Cambridge, UK, USA), anti-snail (ab180714, Abcam), anti-claudin 1(ab15098, Abcam), anti-Sox2 (AB5603, Millipore, Billerica, MA, USA), anti-Bmi1 (ab38295; Abcam), anti-Lin28 (ab46020, Abcam), anti-Nanog (ab62734, Abcam), HIF-1α (ab65979, Abcam) and anti-actin (Sigma, A2066). Actin was used as a loading control. HRP-conjugated secondary goat anti-rabbit or goat anti-mouse (Santa Cruz, Dallas, TX, USA) antibodies were applied to the membranes, which were then developed using a ECL chemiluminescence kit (Pierce, Waltham, MA). After exposure, films were scanned and quantified using the NIH Image J software.

### RNA isolation and quantitative real-time reverse transcription-PCR (qRT-PCR)

TRIzol reagent (Invitrogen, San Diego, CA, USA) was used to extract total RNA according to the manufacturer’s instructions. Then, cDNA was synthesized by reverse transcription from 2 μg RNA using oligodT primers and SuperScript II reverse transcriptase (Invitrogen). qRT-PCR was performed for RER1, E-cadherin, N-cadherin, vimentin, snail, claudin-1, Sox2, Bmi1, Lin28, Nanog and GAPDH. GAPDH was used as an internal control. Each sample was amplified by SYBR Green reaction mix (Qiagen, Valencia, CA, USA) as three replicates in a 20 μL reaction mixture. Delta-delta CT method (2-∆∆Ct) was used to analyze the results and standard ∆∆CT method was applied to measure the fold change in gene expression. Primers used for qRT-PCR were shown below: RER1: F: CACCAAACAGAACGAGGAAT, R: TACAGACCATAGCCACAAGG; snail: F: CTTCAACTGCAAATACTGCAACAAG, R: GCGTGTGGCTTCGGATGT; E-cadherin: F: ACAGTCACTGACACCAACGATAATC, R: ACTGCTGCTTGGCCTCAAA; N-cadherin: F: TGATCGAGAAAAAGTGCAACAGTAT, R: GGCTGTGTTTGAAAGGCCATA; vimentin: F: GCCAGGCAAAGCAGGAGTC, R: GGGTATCAACCAGAGGGAGTG; claudin-1: F: GCTTCATTCTCGCCTTCCT, R: TGACAGCCATCCTCATCTTC; Sox2: F: TGCACCGCTACGACGTGAGC, R: GCCCTGGAGTGGGAGGAAGA; Bmi1: F: CCAGCAAGTATTGTCCTATT, R: CATTCACCTCCTCCTTAGAT; Lin28: F: AAAGGAGACAGGTGCTAC, R: ATATGGCTGATGCTCTGG; Nanog: F: AGTTGGACAGGGAGATGGC, R: AACCTTCCTTGCTTCCACG: HIF-1α: F: GCTTGGTGCTGATTTGTG, R: TGTTTGTTGAAGGGAGAA. GAPDH (internal control gene) primer: F: ACAACTTTGGTATCGTGGAAGG, R: GCCATCACGCCACAGTTTC.

### Cell growth and proliferation assays

Cell proliferation was tested using Cell Counting Kit-8 (Dojindo, Kumamoto, Japan) according to the manufacturer’s instruction. CCK-8 solution was added to each well and incubated for 2 h at 37 °C and 5% CO_2_. The absorbance was measured at 450 nm using a microplate spectrophotometer (Molecular Devices, Sunnyvale, CA, USA). BrdU Cell Proliferation Assay Kit (BioVision Inc.) was employed and performed according to the manufacturer’s instruction. BrdU solution was added into each well and incubated at 37 °C for 3 h and then detected by an enzyme-linked immunosorbent assay.

### Colony formation assay

Colony formation was performed in 6-cm culture dishes. Totally 1000 cells were seeded per dish and cultured for 10 days. Colonies were fixed in methanol for 10 min and stained with 0.1% crystal violet for 1 h at room temperature. The number of colonies was counted by visual estimation.

### Transwell invasion assay

Cells underwent starvation in serum-free DMEM for 24 h and then seeded in the upper chamber of transwell containing 0.2 mL of serum-free medium and Matrigel (BD Bioscience). In the lower chamber, DMEM supplemented with 10% FBS was added. Cells were stained by 0.1% crystal violet and counted after 24 h incubation.

### Wound healing assay

Mechanical scratch was performed for migration test using a sterile serum tube. Cells were cultured for 24 h and then the areas were observed to determine their scratch healing.

### Cell cycle analysis

After knockdown or overexpression of RER1, cells were stained by propidium iodide at room temperature for 30 min. The results were analyzed using a Calibur flow cytometry (BD Biosciences, Franklin Lakers, USA).

### In vivo animal study

A xenograft nude mouse model was used for tumor formation. The animal experiments were approved by Fujian Provincial Hospital, Fujian Medical University (#20160855). 4-week-old BALB/c nude male mice were employed. PANC-1-pSin-vec and PANC-1-pSin-RER1 cells (1 × 10^6^) were subcutaneously injected into the flanks of each nude mouse. Tumor volumes (mm^3^) were measured every 5 days. Mice were sacrificed after 25 days and tumors were resected and paraffin-embedded for further Ki-67 staining.

For lung colonization, PANC-1-pSin-vec and PANC-1-pSin-RER1 cells (1 × 10^6^) were injected into each nude mouse via tail vein, and the incidence of lung metastasis was detected by staining lung sections with standard hematoxylin and eosin (H&E).

### Tumorsphere formation

PANC-1 cells stably transfected with pSin-RER1 or pSin-vec, respectively, were used for tumorsphere formation. Ultra-low adhesion 6-well plates was used and 1 × 10^4^ cells were plated per well, supplemented with 2 mL StemXVivo serum-free tumorsphere media (CCM012, R&D Systems, Minneapolis, MN, USA), 2 U/mL heparin (Sigma) and 0.5 g/mL hydrocortisone (Sigma). Cells were incubated at 37 °C in 5% CO_2_ for 7–10 days. Tumorspheres were then counted under microscope and images were taken at 40× magnification.

### Luciferase reporter assay

Luciferase activity of RER1 promoter was detected in PANC-1 cells in normoxic (21% O_2_) or hypoxic (1% O_2_) conditions. Cells were transfected with RER1 luciferase reporter and pSin-HIF-1α or empty vector pSin-vec by Vigofect reagent (Vigorous Biotechnology, Beijing, China). To clone the constructs of RER1 luciferase reporter, primers for RER1 promoter were shown as below: for RER1 forward primer, CTACTCGAGAAAAGAGAGAGAGAGAAGAAAACTAG, reverse primer, CTAAAGCTTTCCAACCATTTCCGCTTCCGGCGCAGACGCG. For RER1 mutant:forward primer, ACAGTGGAGCGCCCTTACGCACCACCAAAG, reverse primer, CTTTGGTGGTGCGTAAGGGCGCTCCACTGT. The promoter fragments were then sub-cloned into pGL3 Basic luciferase reporter plasmid (Promega). For each well, 10 ng pRL-TK (Promega) was transfected together as an internal control. After 24 h of transfection, cells were harvested and luciferase activity was measured using the Dual-Luciferase Reporter Assay System (Promega, Madison, USA). The results were expressed as the ratio of firefly to Renilla luciferase activity.

### Statistical analysis

Statistical analyses were performed using SPSS Statistics 16.0 (IBM Chicago, IL, USA). χ2 test, two-tailed Student’s t test and two-way ANOVA were used for analysis of differences. Patient survival was analyzed using the Kaplan–Meier method. For all tests, a value of *P* < 0.05 was considered to be statistically significant.

## Results

### RER1 is upregulated in PC tissues and is correlated with poor survival outcome

**Fig. 1:**
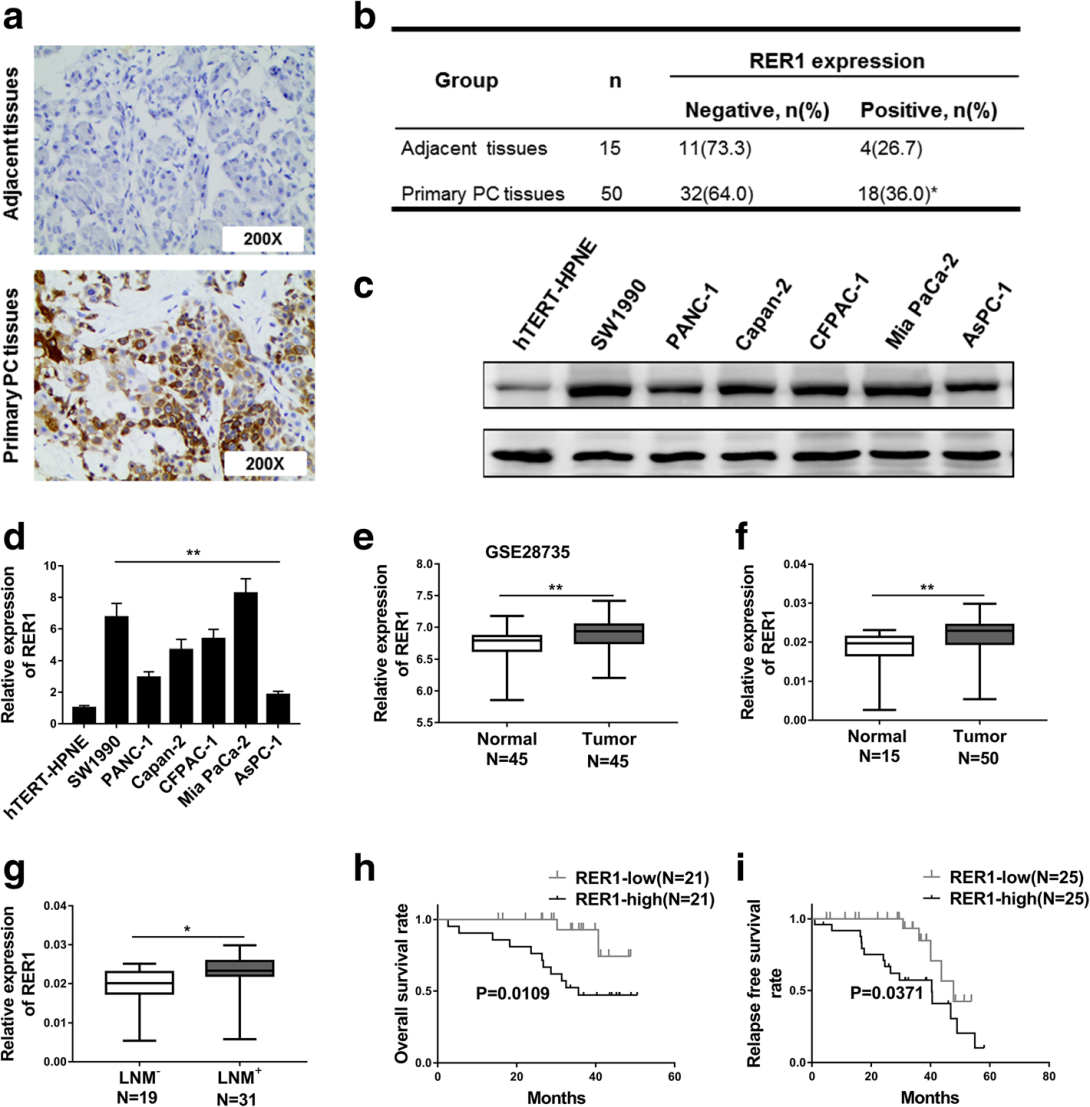
RER1 is upregulated in pancreatic cancer tissues and correlated with poor survival outcome. (**a** and **b**) IHC analysis of RER1 expression levels in human primary PC specimens and non-tumorous pancreas specimens. The representative pictures were shown at 200× magnification. (**c** and **d**) Western blotting and qRT-PCR analysis were performed to test the expression of RER1 in six PC cell lines (AsPC-1, Mia PaCa-2, SW1990, Capan-2, CFPAC-1, and PANC-1) and a normal human pancreatic ductal cell line (hTERT-HPNE). (**e**) The expression level of RER1 was significantly increased in PC tissues compared with normal tissues from GSE28735. (**f**) qRT-PCR analysis of RER1 expression in 50 PC tissues and 15 normal human pancreas tissues (shown as log10 (2-ΔCT)). (**g**) qRT-PCR analysis of RER1 expression in 31 PC tissues from lymph node metastasis (LNM+) patients compared to 19 PC tissues from lymph node metastasis free (LNM-) patients (**h**) Kaplan–Meier’s analysis of the correlation between RER1 expression and the overall survival of PC patients. (**i**) Kaplan–Meier’s analysis of the correlation between RER1 expression and the relapse free survival of PC patients. *P < 0.05; **P < 0.01 (χ2 test for b, student’s *t*-test for others)

To evaluate the role of RER1 in PC, we firstly examined the expression level of Rer1 in PC and adjacent normal tissues (Fig. 1a). 64% of primary PC tissues showed negative Rer1 expression, while 36% showed positive expression (*n* = 50). In adjacent normal tissues, only 26.7% were positive, while 73.3% were negative (*n* = 15, Fig. 1b). This result indicated that Rer1 might be related to PC. Then we further tested Rer1 expression in 6 PC cell lines (AsPC-1, Mia PaCa-2, SW1990, Capan-2, CFPAC-1 and PANC-1) and one normal human pancreatic ductal cell line (hTERT-HPNE). Higher level of Rer1 expression was observed in the 6 PC cell lines compared to hTERT-HPNE (Fig. 1c, d). By analyzing 45 matching pairs of PC from GSE28735, higher Rer1 expression level was shown in tumor compared to adjacent tissues (Fig. 1e). The mRNA levels of RER1 in the 50 PC tissues and 15 adjacent normal tissues were determined by qRT-PCR, which demonstrated a significantly higher RER1 mRNA level in tumor tissues than adjacent tissues (Fig. 1f). We further tested 31 PC tissues from PC patients with or without lymph node metastasis (LNM). Patients with LNM showed higher mRNA level of RER1 in PC compared to patients without LNM (Fig. 1g). These results indicated that RER1 might play a critical role in the development and progression of PC. Thus to explore the importance of RER1 expression in PC progression, we performed Kaplan–Meier’s analysis to determine the correlation between RER1 expression and survival rate of PC patients. We found that patients with higher RER1expression level showed worse overall survival rate and relapse free survival rate compared to those with low RER1 expression (Fig. 1h, i).

### RER1 promotes oncogenicity of PC cells in vitro and in vivo

**Fig. 2:**
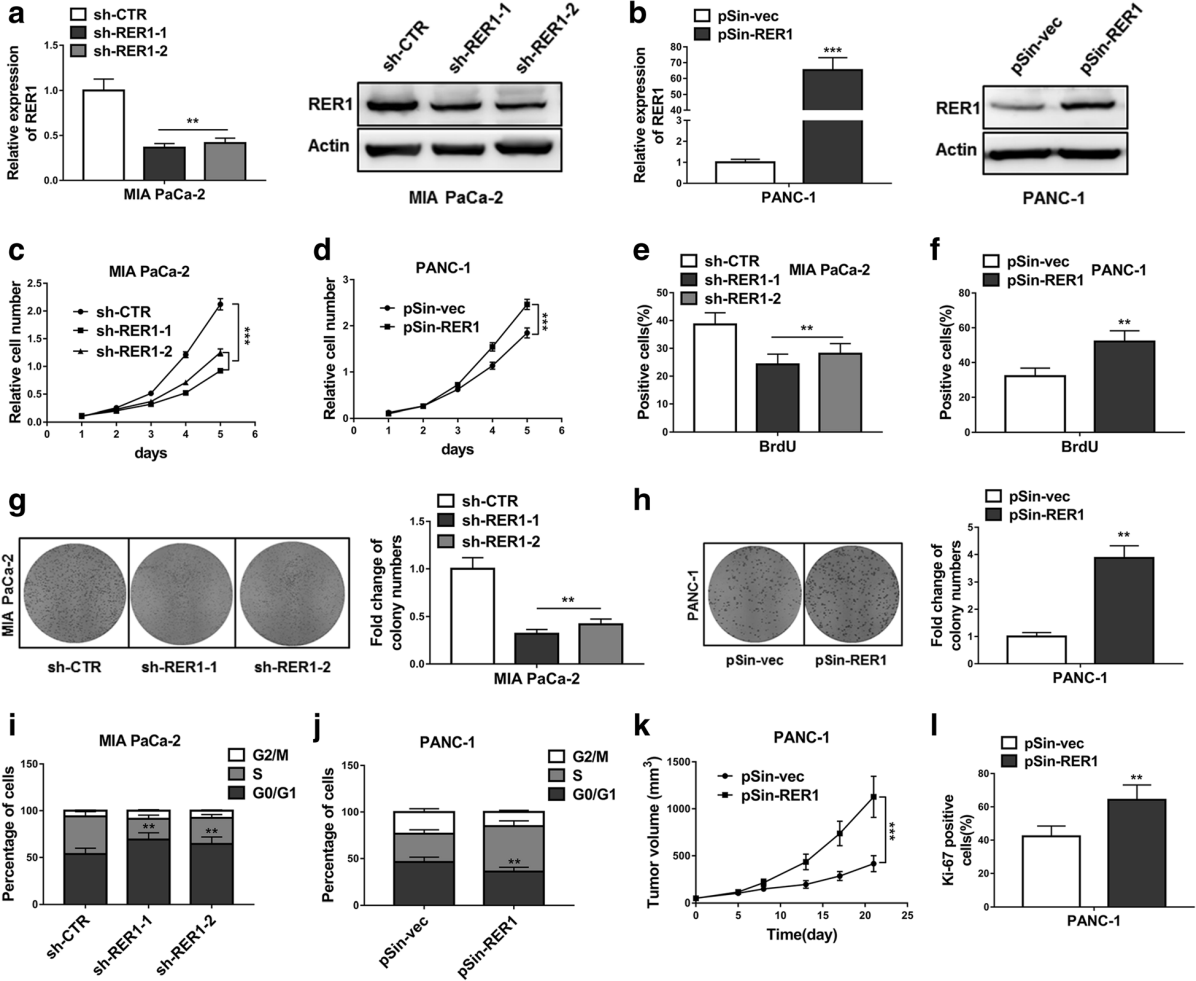
RER1 promotes oncogenicity of PC cells in vitro and in vivo. (**a**) Overexpression or knockdown of RER1 in Mia PaCa-2 cells were determined by qRT-PCR and Western blotting. (**b**) Overexpression or knockdown of RER1 in PANC-1 cells were determined by qRT-PCR and Western blotting. (**c**) CCK-8 assay showed cell survival of RER1 overexpression or knockdown Mia PaCa-2 cells. (**d**) CCK-8 assay showed cell survival of RER1 overexpression or knockdown PANC-1 cells (**e** and **f**) The percentages of BrdU positive Mia PaCa-2 and PANC-1 cells were determined. (**g** and **h**) Colony formation assay was conducted to assess cell survival of Mia PaCa-2 and PANC-1 cells (**i** and **j**) Cell cycle analysis of Mia PaCa-2 and PANC-1 cells were assessed by flow cytometry after staining with PI. The percentages of cells in three phases are shown in histogram. (**k**) Tumor growth curve of PANC-1-pSin-vec and PANC-1-pSin-RER1 cells implanted into the flank of male nude mice. (l) Cell proliferation was measured by Ki-67 staining assay. The data represent the mean ± SD from three independent experiments. *P < 0.05; **P < 0.01; ***P < 0.001 (Two-way ANOVA for c and d, student’s *t*-test for others)

Next, we sought to determine whether RER1 participated in the proliferation of PC cells. Mia PaCa-2 cell lines stably transfected with RER1 shRNAs (sh-RER1–1 and sh-RER1–2) or empty vector (sh-CTR) were established (Fig. 2a). In addition, stable RER1 overexpression was established by stable transfection of RER1 plasmid (pSin-RER1) into PANC-1 cells (Fig. 2b). Both mRNA and protein levels of RER1 were measured. Efficient knockdown of RER1 in Mia PaCa-2 cells led to significantly decreased cell proliferation, while overexpression of RER1 in PANC-1 cells promoted cell growth (Fig. 2c, d). Same observations were obtained by BrdU staining (Fig. 2e, f), suggesting the important function of RER1 in cell proliferation. We next assessed the colony formation ability of these cell lines and found that, knockdown of RER1 remarkably reduced cell survival whereas overexpression of RER1 significantly promoted it (Fig. 2g, h). We next investigated whether the function of RER1 in cell proliferation could affect cell cycle. Lower RER1 expression led to prolonged G0/G1 phases, whereas higher RER1 expression reduced cells in G0/G1 phases and increased cells in S phase (Fig. 2i, j). After confirming the positive role of RER1 in cell proliferation, we implanted PANC-1-pSin-vec and PANC-1-pSin-RER1 cells in to the flanks of male nude mice, and observed that RER1 overexpression markedly promoted tumor growth (Fig. 2k, l). These data suggested RER1 as an essential regulator in PC progression.

### RER1 enhances metastasis and stem-cell like behavior of PC cells

**Fig. 3:**
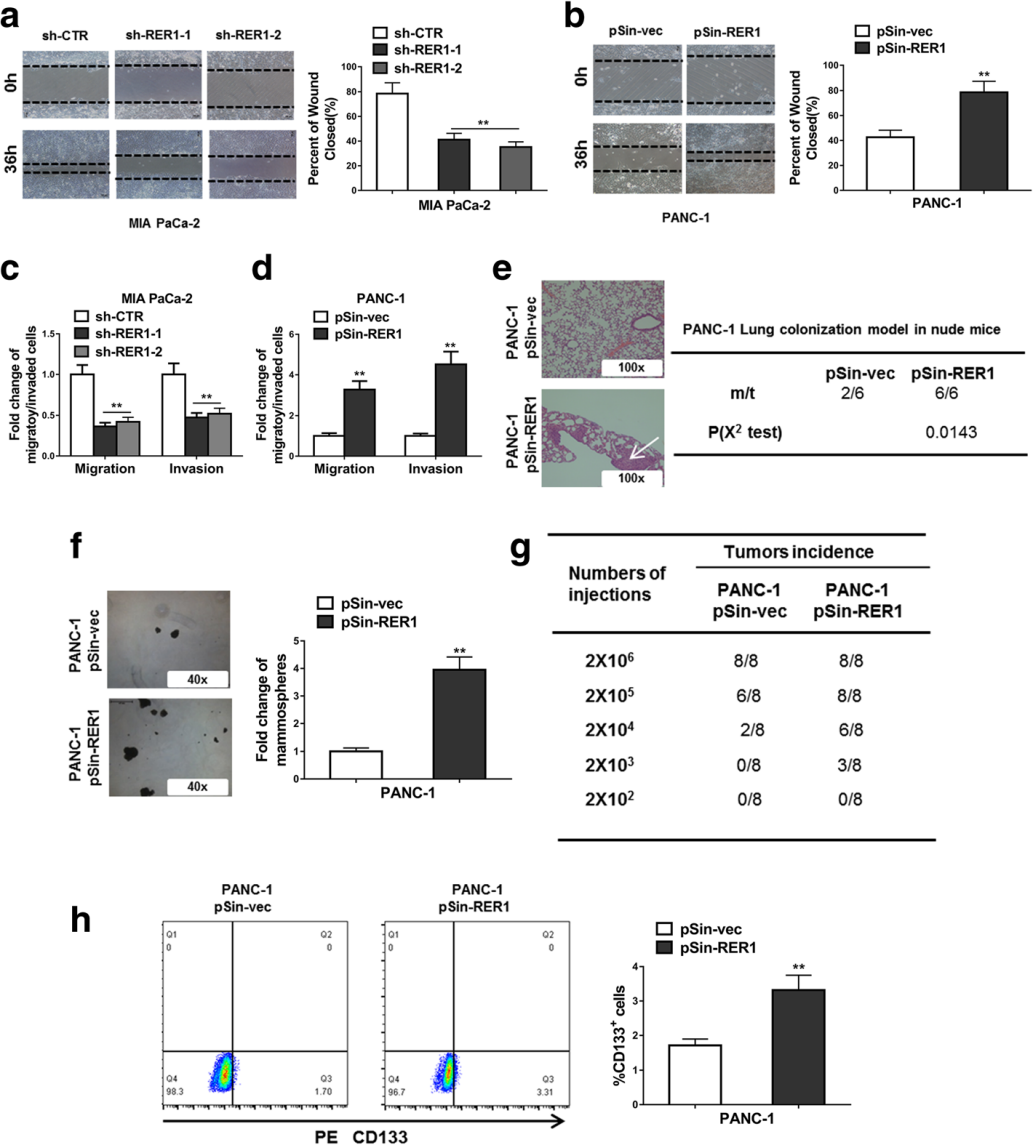
RER1 enhances metastasis and stem-cell like behavior of PC cells. (**a** and **b**) Knockdown of RER1 significantly reduced migration of Mia PaCa-2 cells and overexpression of RER1 significantly increased migration of PANC-1 cells (scratch wound assay). (**c**) Transwell assays were performed to determine the migration and invasion of RER1 overexpression or knockdown Mia PaCa-2 cells. (**d**) Transwell assays were performed to determine the migration and invasion of RER1 overexpression or knockdown PANC-1 cells. (**e**) H&E staining of the metastatic nodules in the lung of RER1 overexpression or knockdown PANC-1 cells. Tail vein injection of PANC-1 cells into nude mice (100X scale bars) was performed and the incidence of lung metastasis in the mice was counted. (**f**) Tumorsphere formation of RER1 overexpression or knockdown PANC-1 cells was performed. The total tumorsphere numbers in each well were counted and images were taken at 40× magnification. (**g**) Tumor incidence of RER1 overexpression or knockdown PANC-1 cells was counted. Cells were injected into the flank of mice with limiting dilutions as indicated. The number of tumors formed in each group was counted after 4 weeks. (**h**) CD133 positive cell population in RER1 overexpression or knockdown PANC-1 cells was determined by flow cytometry. Results are presented in histogram. *P < 0.05; **P < 0.01 (χ^2^ test for e and g, student’s *t*-test for others)

The fact that RER1 expression was higher in patients with LNM raised the possibility that RER1 was involved in PC metastasis. Scratch wound assay showed that knockdown RER1 significantly repressed migration of Mia PaCa-2 cells, whereas overexpression of RER1 dramatically increased cell migration of PANC-1 (Fig. 3a, b). In addition, according to results from the transwell invasion assay, reduced RER1 expression level caused reduced migration and invasion. On the other hand, overexpression of RER1 significantly promoted cell migration and invasion (Fig. 3c, d). We further injected the RER1 overexpressing PANC-1 cells and control cells into nude mice and counted the incidence of lung metastasis. The results demonstrated that RER1 overexpression promoted lung colonization of PANC-1 cells (Fig. 3e). In addition, RER1 also increased the ability of tumorsphere formation of PANC-1 cells (Fig. 3f). When the same number of cells were injected, RER1-overexpressing PANC-1 cells showed higher tumor incidence rate than control cells (Fig. 3g), indicating RER1 was able to enhance tumorigenesis of PANC-1 cells. By staining PANC-1 cells with the stem cell marker CD133, we found that RER1 overexpression resulted in increased number of CD133-positive cells compared to control (Fig. 3h). Therefore, RER1 exerted its functions in not only promoting cell proliferation but also increasing CSC-like phenotype.

### RER1 promotes PC malignance through regulating epithelial-mesenchymal transition (EMT) and CSC pathway

**Fig. 4:**
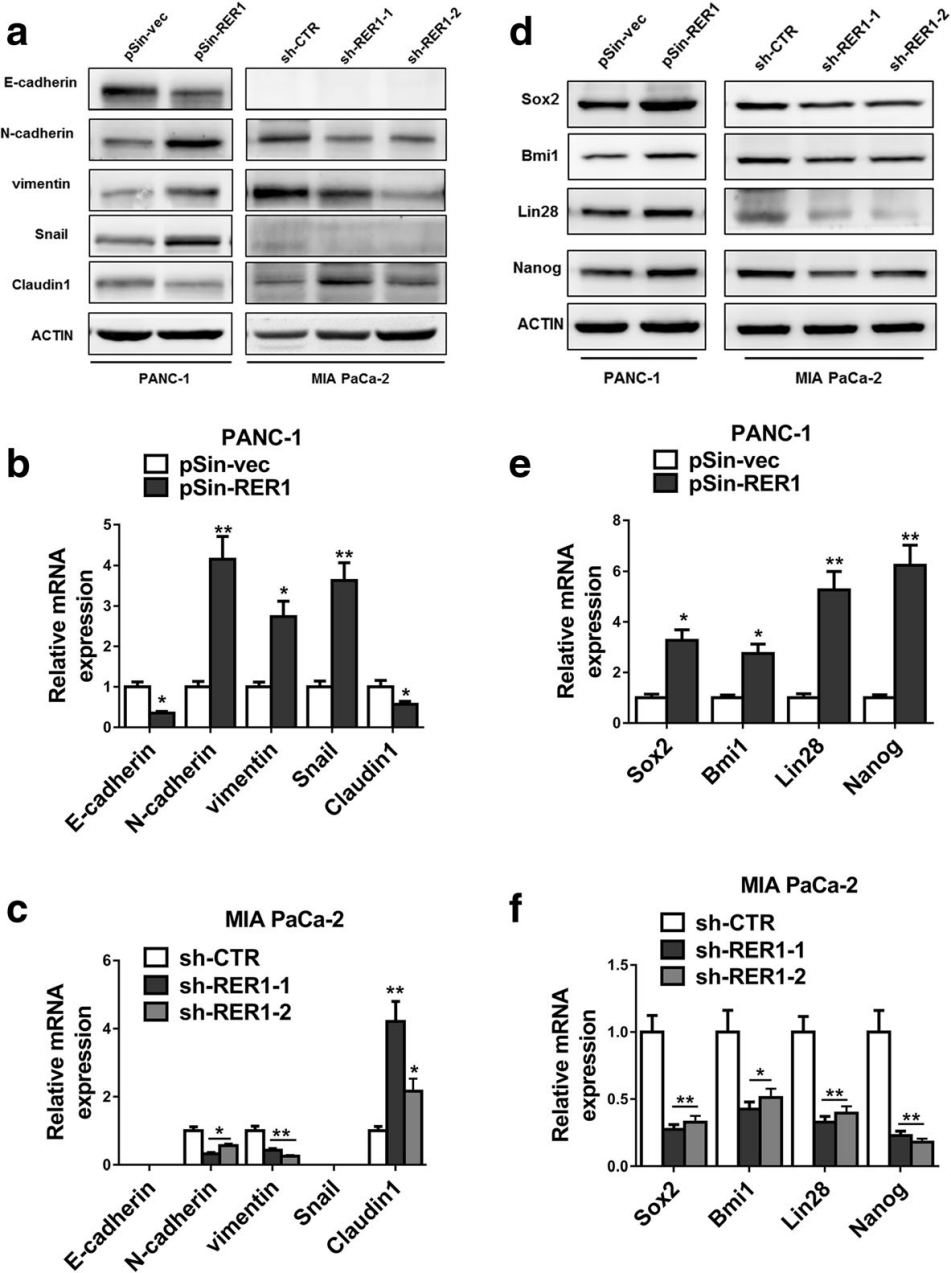
RER1 promotes PC malignance through regulation of EMT and CSC pathway. (**a**-**c**) RER1 overexpression or knockdown PANC-1 and MIA PaCa-2 cells were subjected to Western blot and qRT-PCR analyses of E-cadherin, N-cadherin, vimentin, snail, claudin-1 and actin. (**d**-**f**) RER1 overexpression or knockdown PANC-1 and MIA PaCa-2 cells were subjected to Western blot and qRT-PCR analyses of Sox2, Bmi1, Lin28, Nanog and actin. One representative of at least three independent experiments with similar results is shown. *P < 0.05; **P < 0.01 (Student’s *t*-test)

To further investigate the involvement of RER1 in the progression of PC and CSCs, we firstly tested several EMT markers including E-cadherin, N-cadherin, vimentin, snail and claudin-1, and stem cell markers including Sox2, Bmi1, Lin28 and Nanog. We found that knockdown of RER1 led to remarkably increased claudin-1 and decreased N-cadherin, vimentin and snail expressions (Fig. 4a, b, c). In contrast, overexpression of RER1 upregulated N-cadherin, vimentin and snail, whereas downregulated E-cadherin and claudin-1. These results suggested that RER1 might promote the metastasis of PC through enhancing EMT. In addition to EMT, markers for stemness properties including Sox2, Bmi1, Lin28 and Nanog were significantly repressed in RER1 knockdown cells compared to control cells, whereas overexpression of RER1 promoted the expression of these stemness markers (Fig. 4d, e, f). These data indicated that RER1 might positively regulate EMT and CSCs to enhance the tumorigenesis and metastasis of PC.

### Hypoxia-inducible factor (HIF)-1α regulates RER1

**Fig. 5:**
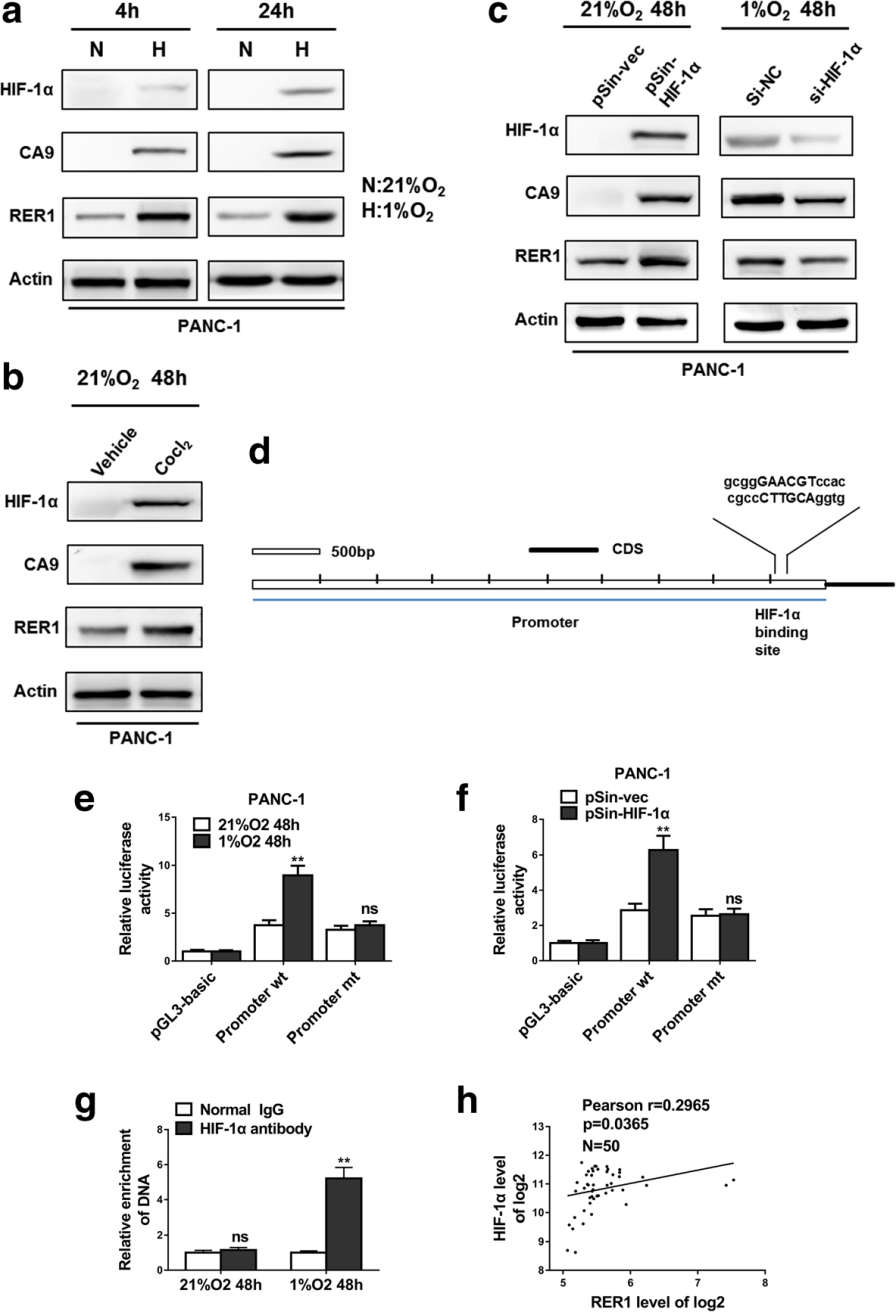
HIF-1α regulates RER1 expression. (**a**) PANC-1 cells were incubated in normoxic or hypoxic condition for the indicated time. RER1, CA9 and HIF-1α expression was determined by Western blot. N: 21% O_2_; H: 1% O_2_. (**b**) Western blot analysis of RER1, CA9 and HIF-1α in PANC-1 cells treated with CoCl_2_ for 48 h under normoxic culture. (**c**) The relationship of RER1, CA9 and HIF-1α expression, determined by Western blot. In normoxia, PANC-1 cells were transiently transfected with HIF-1α plasmid (pSin-HIF-1α) or empty vector (pSin-vec); or HIF-1α was depleted by siRNA under hypoxia for 48 h. (**d**) Bioinformatic analysis of predicted binding sites for HIF-1α in the ~ 3-kb regulatory region upstream of the RER1 start codon. (**e**) Luciferase activity of RER1 promoter. PANC-1 cells were transfected with the wide type promoter sequence of RER1 (Promoter wt) or mutant promoter sequence of RER1 (Promoter mt) and incubated in normoxic (21% O_2_) or hypoxic (1% O_2_) conditions. (**f**) Analysis of promoter activity in PANC-1 cells after transfection of plasmid (pSin-HIF-1α) or empty vector (pSin-vec) under normoxia. (**g**) ChIP assays of PANC-1 cells in normoxic or hypoxic condition. qPCR was carried out with specific primers for promoter sequence of RER1. The histogram shows the fold change of relative enrichment to the input. (**h**) Pearson’s analysis shows correlation between RER1 and HIF-1α levels in 50 PC samples. The data represent the mean ± SD from three independent experiments. *P < 0.05; **P < 0.01

Next, we employed rVista 2.0 (https://rvista.dcode.org/instr_rVISTA.html) and predicted hypoxia-inducible factor (HIF)-1α as a potential transcription factor regulating RER1. In the case of 21% (normoxia) and 1% (hypoxia) of O_2_, induced HIF-1α expression, verified by increased CA9 expression, elevated RER1 protein level (Fig. 5a). Under 21% O_2_, induced HIF-1α (and downstream CA9) expression by CoCl_2_ and overexpression of HIF-1α both promoted RER1 protein level, whereas knockdown HIF-1α by transfection of si-HIF-1α decreased RER1, as well as CA9, expression compared to control (Fig. 5b, c). We next predicted the possible binding sites of HIF-1α on RER1 promoter region, and found that HIF-1α might bind to the ~ 3-kb regulatory region upstream of the RER1 start codon (Fig. 5d). We therefore constructed a mutation in this region RER1 (promoter mt) which could disrupt HIF-1α binding to RER1. As a consequence, induced or overexpressed HIF-1α significantly promoted the transcriptional activity of RER1 but not the mutated version (Fig. 5e, f). Using HIF-1α antibody, DNA segment enriched by HIF-1α was detected by specific RER1 primers. Under 1% O_2_, significantly enriched amount of HIF-1α antibody was found on RER1 compared to IgG control (Fig. 5g). Moreover, Pearson correlation analysis revealed that RER1 and HIF-1α were significantly correlated in the 50 PC samples (Fig. 5h).

### Hypoxia-induced PC progression and CSC phenotype are partially dependent on RER1

**Fig. 6:**
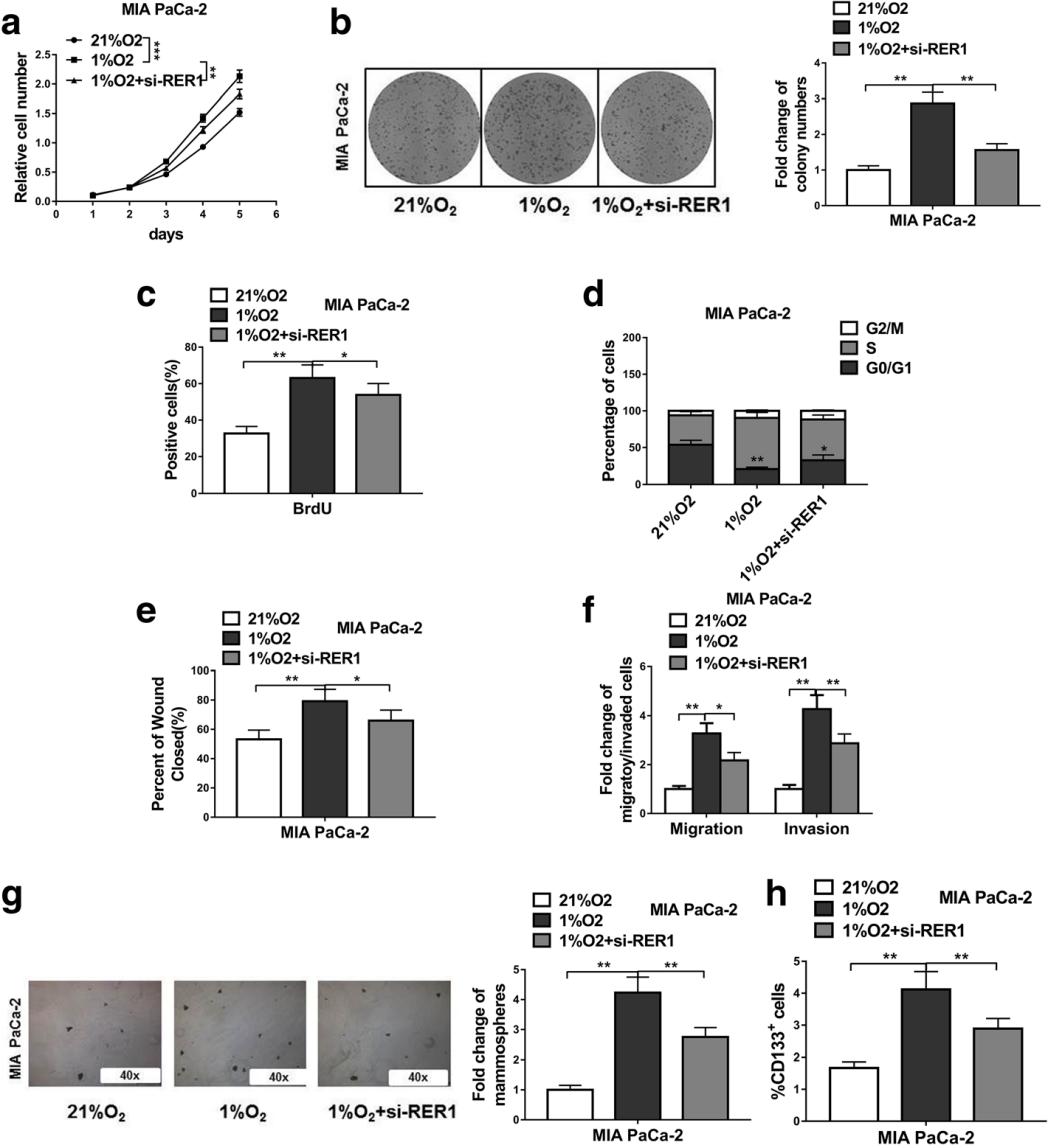
Hypoxia induced PC progression and CSC phenotype are partially dependent on RER1. (**a**-**c**) CCK-8, colony formation and BrdU incorporation assays show viability of MIA PaCa-2 cells under normoxic culture, hypoxic culture or hypoxic culture with RER1 siRNA transfection (21%O_2,_ 1%O_2_ or 1%O_2_ + si-RER1). (**d**) Cell cycle analysis of MIA PaCa-2 cells under normoxic culture, hypoxic culture or hypoxic culture with RER1 siRNA transfection (21%O_2,_ 1%O_2_ or 1%O_2_ + si-RER1) were assessed by flow cytometry after staining with PI. (**e**-**f**) Scratch wound assay and Transwell assays were performed to determine the migration and invasion of MIA PaCa-2 cells under normoxic culture, hypoxic culture or hypoxic culture with RER1 siRNA transfection (21%O_2,_ 1%O_2_ or 1%O_2_ + si-RER1). (**g**) Tumorsphere formation of MIA PaCa-2 cells under normoxic culture, hypoxic culture or hypoxic culture with RER1 siRNA transfection (21%O_2,_ 1%O_2_ or 1%O_2_ + si-RER1). (**h**) CD133 positive cell population in MIA PaCa-2 cells under normoxic culture, hypoxic culture or hypoxic culture with RER1 siRNA transfection (21%O_2,_ 1%O_2_ or 1%O_2_ + si-RER1) was determined by flow cytometry. Results are presented in histogram. The data represent the mean ± SD from three independent experiments. *P < 0.05; **P < 0.01; ***P < 0.001 (Two-way ANOVA for a, student’s *t*-test for others)

When Mia PaCa-2 cells were cultured under hypoxic condition, its proliferation and colony formation ability were significantly increased compared to normoxic culture (Fig. 6a, b, c). However, knockdown of RER1 repressed cell proliferation and colony formation ability under hypoxic condition, which was likely caused by increased percentage of cells in S phase under hypoxic condition whereas knockdown of RER1 led to increased number of cells in G0/G1 phases (Fig. 6d). To further explore the crucial role of RER1 expression under hypoxic condition, si-RER1 was transfected into cells, which significantly repressed migration and invasion compared to control siRNA (Fig. 6e, f). These data suggested that hypoxia-induced cell migration and invasion might partially be regulated by RER1. We further tested the sphere formation ability of Mia PaCa-2 cells. As expected, hypoxia enhanced the number of spheres, which was reduced by RER1 knockdown (Fig. 6g). The percentage of CD133-positive cells were also reduced by RER knockdown compared to control cells under hypoxic condition (Fig. 6h), suggesting hypoxia-induced stemness may be mediated through RER1 action.

## Discussion

In mammalians, most previous studies have only focused on the functions of RER1 in γ-secretase complex assembly and retention in the ER [17–19]. Inactivation or low expression of RER1 could lead to the escape of unassembled acetylcholine receptor α-subunits from the ER and subsequent degradation [20]. Recent studies have revealed that RER1 may be also related to abnormal accumulation of α-synuclein in the ER, which is a critical mechanism of Parkinson disease [21]. However, no previous study has demonstrated the function of RER1 in cancer. The present study is the first report showing the importance of RER1 during PC progression. Here, we demonstrated that PC cell lines showed higher expression of RER1 compared to hTERT-HPNE. RER1 expression levels were higher in PC tumor tissues than adjacent normal tissues, indicating RER1 may play a positive role in PC progression. Advanced stage of PC commonly shows lymph node metastasis. In these patients with LNM, RER1 expression level in PC tissues was higher than in patients without LNM, indicating RER1 may enhance metastasis. Therefore, high RER1 expression may be related to poor prognosis, which was evidenced by Kaplan–Meier’s analysis demonstrating higher rate of relapse and lower rate of survival in patients with higher RER1 expression.

Our further in vitro experiments tested the effects of RER1 on proliferation and colony formation of PC cell lines, indicating RER1 plays an important role in PC cell growth. This effect may be caused by the changes in cell cycle. It seems that the existence of RER1 promotes cells to enter S phase, whereas reduced RER1 expression causes G0/G1 arrest. The mechanism underlying this phenomenon could be complex. A previous study reported that Charcot-Marie-Tooth disease was related to the retention of a peripheral myelin protein 22 (PMP22) mutant regulated by RER1 and calnexin [22]. The retention of PMP22 mutant was suggested to induce ER stress and trigger the apoptosis of Schwann cells, as ER stress is not only associated with cell apoptosis but also cell cycle arrest [23, 24]. However, in the current study, RER1 overexpression may enhance the retention of proteins that promote cell cycle. Further studies are necessary to identify the specific factors involved in this process. Nevertheless, in this study, RER-induced cell cycle changes were able to enhance tumorigenesis of PANC-1 cells.

According to the clinical data demonstrating patients with LNM had higher RER1 expression level than patients without LNM, it could be speculated that RER1 might play a role in metastasis of PC cells. We used knockdown RER1 cell lines to verify that RER1 significantly regulated migration and invasion of PC cells. Intriguingly, mice injected with RER1-overexpressing PANC-1 cells showed lung colonization, whereas only about 33% of mice injected with control PANC-1 cells exhibited lung colonization. Taken together, RER1 may enhance PC progression by promoting PC cell growth and metastasis. A recent finding reported that a gain-of-function mutant p53, found in 75% of pancreatic adenocarcinoma, highly drived tumor metastasis via uridine 5′-diphosphatase in the ER [25]. This study has prompted us with a clue, that RER1-induced metastasis of PC cells may be mediated by such proteins in the ER.

Using viral infection to introduce stable overexpression or knockdown of genes in cancer cell lines is a common and efficient strategy in various studies, including our own. However, cautions should be taken on the nature of established cell clones, since individual random clones obtained from a cell line can display phenotypical differences. To address this issue, significant efforts were made during our investigation to improve our experimental protocol, which yielded markedly high infection efficiency (~ 80%), to ensure that cell populations used in this study were indeed polyclonal after puromycin selection. Furthermore, we chose at least two shRNAs for the knockdown of the target gene, which is also widely accepted procedure in studies involving shRNAs, to rule out possibility of spontaneous or off-target phenomenon. These considerations are critical to demonstrate that it is indeed the modulation of the specific gene expression that lead to the change of relative biological effects in our current study.

Over the last decade, our understanding of cancer has expanded. It is widely suggested that CSCs play essential roles in the clonal maintenance of the neoplasm, drug resistance, recurrence and tumor metastasis [26, 27]. Therefore, we further determined whether the enhanced tumorigenesis and metastasis of PC by RER1 were attributable to its functions in CSCs. As expected, RER1 enhanced stemness of PANC-1 cells. In addition, EMT is an important mechanism for metastasis of cancer cells, which allows epithelial cells to differentiate into mesenchymal cells exhibiting increased migratory and invasive properties [28]. We detected several EMT markers in cell lines with RER knockdown or overexpression. The results showed that RER1 upregulated EMT-related genes, indicating RER1 may promote PC metastasis via enhancing EMT. Because we identified that RER1 is capable of inducing stemness of PC cells, it is also possible that RER1 overexpression elicits the conversion of PC cells to CSCs, which undergo EMT to generate circulating tumor cells. Numerous previous studies have suggested that CSCs serve as a crucial factor of metastasis, which may even determine the occurrence of metastasis [27]. However, to clarify the correlation between EMT and CSC, further studies are needed.

Here, we have identified that HIF-1α binds to a ~ 3 kb regulatory region upstream of the RER1 start codon to regulate its expression. In tumors, impaired oxygen balance leads to a hypoxic environment, causing rapid proliferation of cancer cells [29]. Among the signaling pathways adapted to this harsh microenvironment, HIF-1α activation has been extensively reported [29–31] and implicated in tumor invasiveness and metastasis [32]. In this study, under hypoxic condition, induced expression of HIF-1α increased RER1 expression, demonstrating that the hypoxia-induced PC cell proliferation, invasion and migration may function through upregulating RER1 by HIF-1α. Accumulating evidences have shown that HIF-1α is required in maintaining the stemness of CSCs [4, 33]. Knockdown or inhibition of HIF-1α abrogates CSCs in hematological malignancies [34]. It is possible that these functions of HIF-1α are mediated through RER1 action.

## Conclusions

In conclusion, this study shows that RER1 expression is related to poor outcomes of PC. Our study is the first to report the important roles of RER1 in PC progression. RER1 is able to enhance EMT and increase stemness of CSCs. Furthermore, we have identified HIF-1α as an upstream regulator controlling RER1 expression, and the functions of HIF-1α may be partially dependent on RER1. Diminished migratory and invasive abilities of PC cells are observed following RER-1 knockdown under hypoxic condition. The present study demonstrates a new mechanism underlying PC progression and provides a potential novel target for PC treatment.

## Abbreviations

COP I: Coat protein I
CSCs: Cancer stem cells
ER: Endoplasmic reticulum
HIF: Hypoxia-inducible factor
PC: Pancreatic cancer
RER1: Retention in Endoplasmic Reticulum 1
